# Community norms of the Muscle Dysmorphic Disorder Inventory (MDDI) among cisgender sexual minority men and women

**DOI:** 10.1186/s12888-021-03302-2

**Published:** 2021-06-08

**Authors:** Jason M. Nagata, Emilio J. Compte, Chloe J. Cattle, Jason M. Lavender, Tiffany A. Brown, Stuart B. Murray, Annesa Flentje, Matthew R. Capriotti, Micah E. Lubensky, Juno Obedin-Maliver, Mitchell R. Lunn

**Affiliations:** 1 Department of Pediatrics, University of California, San Francisco, Box 0110, 550 16th Street, 4th Floor, San Francisco, CA 94158 USA; 2 Eating Behavior Research Center, School of Psychology, Universidad Adolfo Ibáñez, Santiago, Chile; 3 Research Department, Comenzar de Nuevo Treatment Center, Monterrey, Mexico; 4 Military Cardiovascular Outcomes Research Program (MiCOR), Department of Medicine, Uniformed Services University of the Health Sciences, Bethesda, MD USA; 5 The Metis Foundation, San Antonio, TX USA; 6 Department of Psychiatry, University of California, San Diego, San Diego, CA USA; 7 San Diego State University Research Foundation, San Diego, CA USA; 8 Department of Psychiatry and Behavioral Sciences, University of Southern California, Los Angeles, CA USA; 9 Department of Community Health Systems, University of California, San Francisco, San Francisco, CA USA; 10 Alliance Health Project, Department of Psychiatry and Behavioral Sciences, University of California, San Francisco, CA USA; 11 The PRIDE Study/PRIDEnet, Stanford University School of Medicine, Stanford, CA USA; 12 Department of Psychology, San José State University, San Jose, CA USA; 13 Department of Obstetrics and Gynecology, Stanford University School of Medicine, Stanford, CA USA; 14 Department of Epidemiology and Population Health, Stanford University School of Medicine, Stanford, CA USA; 15 Division of Nephrology, Department of Medicine, Stanford University School of Medicine, Stanford, CA USA

**Keywords:** Muscle dysmorphia, Muscle dysmorphic disorder, Body image, Body dissatisfaction, Body dysmorphia, Gay, Lesbian, Bisexual, Pansexual, Polysexual, Homosexual, Sexual minority, LGBTQ

## Abstract

**Background:**

Representing the pathological extreme pursuit of muscularity, muscle dysmorphia (MD) is characterized by a pervasive belief or fear around insufficient muscularity and an elevated drive for muscularity. Despite evidence of elevated body image-related concerns among sexual minority populations, little is known about the degree of muscle dysmorphia (MD) symptoms among sexual minorities, particularly based on Muscle Dysmorphic Disorder Inventory (MDDI) scores. The objective of this study was to examine the nature and severity of MD symptoms in cisgender sexual minority men and women and provide community norms of the MDDI for these populations.

**Methods:**

Data from participants in The PRIDE Study, an existing study of health outcomes in sexual and gender minority people from the United States, were examined. Participants included cisgender gay men (*N* = 1090), cisgender bisexual plus (bisexual, pansexual, and/or polysexual) men (*N* = 100), cisgender lesbian women (*N* = 563), and cisgender bisexual plus women (*N* = 507). We calculated means, standard deviations (SD), and percentiles for the MDDI total and subscale scores for cisgender sexual minority men and women. We compared MDDI scores by sexual orientation using linear regression models, both unadjusted and adjusted for sociodemographics.

**Results:**

Overall, the sample was 85.2% White, 3.0% Asian or Pacific Islander, 2.0% Black, 0.5% Native American, 3.9% multiracial, and 6.6% Hispanic/Latino/a. The mean age was 38.6 (*SD* = 14.3) and 69.4% had a college degree or higher. Means (*SD*) for the MDDI total score were 27.4 (7.7) for cisgender gay men, 26.4 (6.4) for cisgender bisexual plus men, 24.3 (6.1) for cisgender lesbian women, and 24.6 (5.5) for cisgender bisexual plus women. There were no significant differences in MDDI scores between cisgender gay and bisexual plus men, or between cisgender lesbian women and bisexual plus women in unadjusted or adjusted models.

**Conclusions:**

These normative data provide insights into the experience of MD symptoms among cisgender sexual minority men and women and can aid researchers and clinicians in the evaluation of MD symptoms and interpretation of MDDI scores in sexual minority populations.

**Supplementary Information:**

The online version contains supplementary material available at 10.1186/s12888-021-03302-2.

## Introduction

Muscle dysmorphia (MD), currently diagnosed using a specifier for Body Dysmorphic Disorder (BDD) in the Diagnostic and Statistical Manual of Mental Disorders, 5th Edition (DSM-5, American Psychiatric Association, 2013), is characterized by the pathological pursuit of muscularity and a preoccupation with one’s body size as not being large enough [1, 2]. The condition is associated with numerous behavioral disturbances, including excessive exercise training, rigid diets, disordered eating patterns, and anabolic steroid use [3–5]. Individuals with MD commonly report that they have little to no control over these behaviors and have been found to exhibit poor insight related to these behaviors [6]. Notably, there is significant comorbidity between MD and various forms of psychopathology, particularly mood and anxiety disorders [6–8], as well as substance use and suicidality [4, 9]. Along with the shame and distress associated with MD, the time-consuming and rigid nature of the common behavioral patterns promotes significant impairments in social and occupational functioning [2, 4]. Cumulatively, these findings emphasize the high degree of distress and impairment than can arise from MD and the importance of characterizing the nature and degree of MD symptoms in at-risk populations.

Although the literature on MD has expanded in recent years, questions still remain regarding how MD symptoms present across various identity dimensions [10]. Specifically, MD was initially characterized in reference to bodybuilding men, and the majority of MD research has utilized small sample sizes of presumably cisgender (i.e.*,* those whose gender identity matches what is commonly associated with the sex assigned to them at birth) heterosexual populations of men [8, 10, 11]. For example, in a recent meta-analysis of 34 studies on MD, only six had samples that included participants who identified as women [12]. Nearly all of these studies neglected to assess or report the sexual orientation of participants, which is particularly problematic given evidence suggesting that gay (versus heterosexual) men are more likely to endorse dissatisfaction with their physical appearance and muscle size/tone [13] and to hold distorted cognitions about the importance of having an “ideal physique” [14, 15]. Preliminary evidence also suggests that both gay (versus heterosexual) men and lesbian (versus heterosexual) women exhibit a greater drive for muscularity [16]. Gay men (presumably cisgender) report being most dissatisfied with their muscularity compared to other body parts, and muscularity is considered important to being perceived as physically attractive [17]. Studies of BDD also have found that sexual minority individuals (presumably cisgender) endorsed a greater number of BDD symptoms than heterosexual individuals [18]. Further, among sexual minority adolescent boys and young men, gay-related rejection sensitivity and sexual orientation concealment were associated with greater BDD symptoms [19]. Notably, there are extremely limited data on MD-related symptoms among individuals with plurisexual identities (i.e.*,* romantic or sexual attraction and/or behavior with members of more than one sex or gender), such as bisexual, pansexual, and polysexual identities, which we refer to as bisexual plus.

With regard to the measures that have been developed to assess and screen for MD, the Muscle Dysmorphic Disorder Inventory (MDDI) [20] has been one of the most widely used. The 13-item questionnaire consists of three subscales focusing on unique components of MD, including drive for size, appearance intolerance, and functional impairment. Functional impairment in particular represents a crucial element of the disorder that certain other measures fail to assess [21]. The MDDI has been psychometrically examined in diverse samples of men – spanning multiple languages, countries, and demographics – with results suggesting that the MDDI scores demonstrate reliability and validity across a variety of populations [5, 9, 22–26]. However, there remains a lack of published normative data for the MDDI, including in at-risk populations, such as cisgender sexual minority people, limiting the interpretability of scores on the measure in certain groups.

In order to address the paucity of MD literature on sexual minorities and to provide insights into the nature and degree of MD symptoms within these populations, the present study examined MDDI norms in community samples of cisgender gay men, cisgender bisexual plus men, cisgender lesbian women, and cisgender bisexual plus women. Further, to characterize differences within the samples of cisgender men and cisgender women reporting different sexual orientations, we conducted both unadjusted and adjusted (for sociodemographic characteristics) comparisons of MDDI mean total and subscale scores in cisgender gay versus cisgender bisexual men, and in cisgender lesbian versus cisgender bisexual women.

## Method

### Study population

Data in this study were drawn from a subsample of participants from The Population Research in Identity and Disparities for Equality (PRIDE) Study who completed the ‘Eating and Body Image’ survey from April 2018 to August 2018. Of the 10,665 participants in The PRIDE Study at that time, 4285 completed the ‘Eating and Body Image’ survey. The PRIDE Study is a large-scale, national (United States), and longitudinal cohort study of sexual and gender minority adults. Specific inclusion criteria were: identification as a sexual and/or gender minority person, living in the United States or its territories, age ≥ 18 years, and ability to read and respond to a questionnaire written in English. Participants were recruited through PRIDEnet (a national network of organizations and individuals to engage sexual and gender minority communities), digital communications (e.g., blog posts, newsletters), distribution of The PRIDE Study-branded promotional items, outreach at conferences and events, social media advertising, and word-of-mouth. Data were collected on a cloud-based, web-responsive, and secure platform accessible from any smartphone, tablet, or computer. Additional details about The PRIDE Study research platform, recruitment, and design are reported elsewhere [27, 28].

For this study, we included cisgender gay men, cisgender bisexual plus men, cisgender lesbian women, and cisgender bisexual plus women. Participants were asked about the sex assigned to them at birth (“What sex were you assigned at birth on your original birth certificate?”), their current gender identity (with the option to indicate more than one), and their sexual orientation (with the option to indicate more than one). Additional file 1 describes the classification rules that were applied to form the final samples. Of the 4285 participants who completed the ‘Eating and Body Image’ survey, the final sample included participants (*n* = 2260) who were classified as cisgender gay men (*N* = 1090), cisgender bisexual plus men (*N* = 100), cisgender lesbian women (*N* = 563), and cisgender bisexual plus women (*N* = 507). No compensation was given for survey completion. This study was approved by the University of California, San Francisco and Stanford University Institutional Review Boards, as well as The PRIDE Study’s Research Advisory Committee and Participant Advisory Committee. All study procedures and methods were carried out in accordance with relevant guidelines and regulations.

### Measures

#### Sociodemographic questionnaire

Sociodemographic information including age, race/ethnicity, country of birth, and education level was self-reported. Body mass index (BMI) was calculated from self-reported weight and height [weight (kg)/height (m)^2^]. Questions also assessed participant history regarding diagnosed BDD and MD. Specifically, participants were asked, “Has a mental health professional or physician ever told you that you have Body Dysmorphic Disorder (BDD)?” and “Has a mental health professional or physician ever told you that you have Muscle Dysmorphia?”

#### Muscle dysmorphic disorder inventory (MDDI)

The MDDI is a 13-item measure that assesses symptoms of muscle dysmorphia [20]. Respondents rate statements on a 1 (*never*) to 5 (*always*) scale. The MDDI includes a total score and three subscale scores: Drive for Size (DFS, 5 items, range 5–25), Appearance Intolerance (AI, 4 items, range 4–20), and Functional Impairment (FI, 4 items, range 4–20). The MDDI has demonstrated strong psychometric properties among college-aged men [20], and the factor structure (as well as invariance), internal consistency, reliability, and convergent validity has been supported in cisgender gay men and lesbian women [29, 30]. For cisgender women in this study, item five *(“I think my chest is too small”*) was modified to specify “chest (muscle)”, so as to not confuse “chest” with breast size [30]. In cisgender gay men, Cronbach’s alpha was 0.77 for the total score, 0.85 for DFS, 0.84 for AI, and 0.84 for FI. In cisgender bisexual plus men, Cronbach’s alpha was 0.69 for the total score, 0.87 for DFS, 0.82 for AI, and 0.78 for FI. In cisgender lesbian women, Cronbach’s alpha was 0.72 for the total score, 0.76 for DFS, 0.81 for AI, and 0.85 for FI. In cisgender bisexual plus women, Cronbach’s alpha was 0.67 for the total score, 0.74 for DFS, 0.80 for AI, and 0.81 for FI.

### Data analysis

Results are presented in terms of percentiles, mean (standard deviation), median (interquartile range [IQR]), and range. In addition, linear regression analyses were used for group comparisons by sexual orientation within genders (cisgender gay men 0, cisgender bisexual plus men 1; cisgender lesbian women 0, cisgender bisexual plus women 1). Given non-normal distributions of the MDDI total score and the DFS and FI subscale scores, these variables were log transformed. Models included MDDI score as the dependent variable and sexual orientation as the independent variable, separately among cisgender men and cisgender women. Adjusted models included age, race, ethnicity, education, and BMI. Two-tailed tests with an adjusted *p*-value (Bonferroni) were set at 0.0125 for statistical significance.

The R statistical environment (RStudio, version 3.6.2) was used to calculate norms, and Stata (StataCorp, version 15.1) was used to conduct regression analyses. For missing values (cisgender gay men: 0.06%; cisgender bisexual plus men: 0.04%; cisgender lesbian women: 0.08%; cisgender bisexual plus women: 0.06%), the mechanism of missing data was examined using the nonparametric test of homoscedasticity from the *MissMech* package [31]; given that in all cases missing mechanisms were consistent with missing completely at random (*p*s > 0.05), data imputation was performed using multivariate imputation by chained equations through the *mice* package [32].

## Results

### Cisgender gay men

Among the 1090 participants who identified as cisgender gay men, the mean age was 42.1 (*SD* = 15.1, range 18–82) years, and the mean BMI was 27.2 (*SD* = 6.3) kg/m^2^ (Table 1). Of the total subsample of cisgender gay men, 85.1% identified as White, 3.2% as Asian or Pacific Islander, 1.9% as Black, 0.7% as Native American, 4.8% as multiracial, and 4.3% as another race. In addition, 7.6% of the participants identified as Hispanic/Latino/a. Among cisgender gay men, 73.3% had a college degree or higher. Overall, 2.0% of participants reported having ever been told by a healthcare provider that they had BDD, and 0.4% reported having been told that they had MD. Norms for the MDDI among cisgender gay men are presented in Table 2.

**Table 1:** Sociodemographic characteristics of cisgender sexual minority men and women from The PRIDE Study

	Total	Cisgender gay men	Cisgender bisexual plus men	Cisgender lesbian women	Cisgender bisexual women
N	2260	1090	100	563	507
**Sociodemographic characteristics**	Mean ± SD / %	Mean ± SD / %	Mean ± SD / %	Mean ± SD / %	Mean ± SD / %
Age, years	38.6 ± 14.3	42.1 ± 15.1	38.0 ± 12.8	38.0 ± 14.3	31.9 ± 9.6
Race					
White	85.2%	85.1%	83.5%	85.4%	85.3%
Asian/Pacific Islander	3.0%	3.2%	7.7%	1.6%	3.1%
Black/African American	2.0%	1.9%	2.2%	1.3%	2.9%
Native American	0.5%	0.7%	0.0%	0.5%	0.2%
Multiracial/Other	3.9%	4.8%	1.1%	4.4%	1.9%
Another Race	5.5%	4.3%	5.5%	6.9%	6.5%
Ethnicity: Hispanic/Latino/a	6.6%	7.6%	3.0%	6.0%	5.9%
Educational attainment					
College degree or higher	69.4%	73.3%	75.0%	56.5%	74.4%
Body mass index (BMI), kg/m^2^	28.1 ± 7.4	27.2 ± 6.5	28.1 ± 7.1	29.1 ± 8.1	29.0 ± 8.4

**Table 2:** Means, standard deviations, medians, interquartile ranges, and percentile ranks for the Muscle Dysmorphic Disorder Inventory (MDDI) total and subscale scores among cisgender gay men (*N* = 1090) and cisgender bisexual plus men (*N* = 100) from The PRIDE Study

	Cisgender gay men (*N* = 1090)				Cisgender bisexual plus men (*N* = 100)			
	MDDI DFS	MDDI AI	MDDI FI	MDDI Total	MDDI DFS	MDDI AI	MDDI FI	MDDI Total
M (SD)	9.9 (4.6)	11.5 (4.3)	6.1 (3.0)	27.4 (7.7)	9.0 (4.2)	11.1 (4.1)	6.4 (2.8)	26.4 (6.4)
Mdn (IQR)	9 (20)	11 (16)	5 (16)	27 (50)	8 (17)	11 (16)	5 (12)	26 (29)
Range	5–25	4–20	4–20	13–63	5–22	4–20	4–16	13–42
Percentile rank								
5	5.0	5.0	4.0	16.0	5.0	5.0	4.0	17.0
10	5.0	6.0	4.0	18.0	5.0	6.0	4.0	18.0
15	5.0	6.0	4.0	20.0	5.0	6.9	4.0	18.9
20	6.0	7.0	4.0	21.0	5.0	7.0	4.0	20.8
25	6.0	8.0	4.0	22.0	6.0	8.0	4.0	21.0
30	7.0	9.0	4.0	23.0	6.0	8.0	4.0	23.0
35	7.0	9.0	4.0	24.0	6.0	8.7	4.0	24.0
40	7.0	10.0	4.0	25.0	7.0	10.0	5.0	25.0
45	8.0	11.0	4.0	26.0	7.0	11.0	5.0	25.6
50	9.0	11.0	5.0	27.0	8.0	11.0	5.0	26.0
55	9.0	12.0	5.0	28.0	8.0	12.0	6.0	27.0
60	10.0	13.0	6.0	28.0	9.0	12.0	7.0	27.4
65	11.0	13.0	6.0	29.0	9.0	13.0	7.0	29.0
70	12.0	14.0	7.0	30.0	10.0	13.0	7.0	30.0
75	13.0	15.0	7.0	31.0	11.0	14.0	8.0	31.0
80	14.0	16.0	8.0	33.0	11.20	15.0	9.0	32.0
85	15.0	17.0	9.0	35.0	12.15	15.2	9.0	34.0
90	17.0	17.0	10.0	38.0	15.0	17.0	10.0	35.0
95	19.0	19.0	12.0	41.0	18.1	18.0	12.1	37.1
99	23.0	20.0	16.11	48.0	22.0	20.0	15.0	40.0

*MDDI*: Muscle Dysmorphic Disorder Inventory, *MDDI DFS* MDDI: Drive for Size subscale, *MDDI AI* MDDI: Appearance Intolerance subscale, *MDDI FI* MDDI: Functional Impairment subscale, *M*: Mean, *SD*: Standard deviation, *Mdn*: Median, *IQR*: Interquartile range

### Cisgender bisexual plus men

Among the 100 participants who identified as cisgender bisexual plus men, the mean age was 38.0 (*SD* = 12.8, range 20–76) years, and the mean BMI was 28.1 (*SD* = 7.1) kg/m^2^ (Table 1). Among cisgender bisexual plus men, 83.5% identified as White, 7.7% as Asian or Pacific Islander, 2.2% as Black, 1.1% as multiracial, and 5.5% as another race. In addition, 3.0% of the participants identified as Hispanic/Latino/a. Among cisgender bisexual plus men, 75.0% had a college degree or higher. Overall, 2.0% of participants reported having ever been told by a healthcare provider that they had BDD, and none reported having been told that they had MD. Norms for the MDDI among cisgender bisexual plus men are presented in Table 2.

### Comparisons between cisgender gay men and cisgender bisexual plus men

In unadjusted linear regression analyses, no significant differences in the MDDI total score (B = − 0.03, 95% CI -0.09-0.03, *p* = 0.358) or the subscales (DFS [B = − 0.09, 95% CI -0.18-0.01, *p* = 0.066], AI [B = − 0.35, 95% CI -1.25-0.55, *p* = 0.442], and FI [B = 0.06, 95% CI -0.02-0.14, *p* = 0.165]) were observed for cisgender bisexual plus men compared to cisgender gay men.

In linear regression analyses adjusted for sociodemographics, no significant differences in the MDDI total score (B = − 0.05, 95% CI -0.10-0.01, *p* = 0.112) or the subscales (DFS [B = − 0.09, 95% CI -0.17-0.00, *p* = 0.041], AI [B = − 0.80, 95% CI -1.60-0.01, *p* = 0.046], and FI [B = 0.05, 95% CI -0.03-0.14, *p* = 0.206]) were observed for cisgender bisexual plus men compared to cisgender gay men.

### Cisgender lesbian women

Among the 563 participants who identified as cisgender lesbian women, the mean age was 38.0 (*SD* = 14.3, range 18–77) years, and the mean BMI was 29.1 (*SD* = 8.1) kg/m^2^ (Table 1). Among cisgender lesbian women, 85.4% identified as White, 1.6% as Black, 1.3% as Asian or Pacific Islander, 0.5% as Native American, 4.4% as multiracial, and 6.9% as another race. In addition, 6.0% of the participants identified as Hispanic/Latino/a. Among cisgender lesbian women, 73.8% had a college degree or higher. Overall, 2.5% of the participants reported having ever been told by a healthcare provider that they had BDD, and none reported having been told that they had MD. Norms for the MDDI among cisgender lesbian women are presented in Table 3.

**Table 3:** Means, standard deviations, medians, interquartile ranges, and percentile ranks for the Muscle Dysmorphic Disorder Inventory (MDDI) total and subscale scores among cisgender lesbian women (N = 563) and cisgender bisexual plus women (*N* = 507) from The PRIDE Study

	Cisgender lesbian women (*N* = 563)				Cisgender bisexual plus women (*N* = 507)			
	MDDI DFS	MDDI AI	MDDI FI	MDDI Total	MDDI DFS	MDDI AI	MDDI FI	MDDI Total
M (SD)	6.4 (2.5)	11.9 (4.1)	6.1 (3.0)	24.3 (6.1)	6.4 (2.4)	12.2 (3.9)	6.1 (2.9)	24.6 (5.5)
Mdn (IQR)	5 (16)	12 (16)	4 (16)	24 (41)	5 (16)	12 (16)	5 (16)	24 (31)
Range	5–21	4–20	4–20	13–54	5–21	4–20	4–20	13–44
Percentile rank								
5	5.0	5.0	4.0	15.2	5.0	6.0	4.0	17.0
10	5.0	6.0	4.0	16.3	5.0	7.0	4.0	18.0
15	5.0	7.0	4.0	18.0	5.0	8.0	4.0	19.0
20	5.0	8.0	4.0	19.0	5.0	9.0	4.0	20.0
25	5.0	9.0	4.0	20.0	5.0	9.0	4.0	21.0
30	5.0	9.0	4.0	21.0	5.0	10.0	4.0	21.0
35	5.0	10.0	4.0	22.0	5.0	10.0	4.0	22.0
40	5.0	11.0	4.0	23.0	5.0	11.0	4.0	23.0
45	5.0	11.0	4.0	23.0	5.0	12.0	4.0	23.0
50	5.0	12.0	4.0	24.0	5.0	12.0	5.0	24.0
55	5.0	13.0	5.0	24.0	5.0	13.0	5.0	25.0
60	5.0	13.0	5.0	25.8	6.0	13.0	6.0	26.0
65	6.0	14.0	6.0	26.0	6.0	14.0	6.0	27.0
70	6.0	15.0	7.0	27.0	7.0	15.0	7.0	27.0
75	7.0	15.0	7.0	28.0	7.0	15.0	7.0	28.0
80	7.0	16.0	8.0	29.0	8.0	16.0	8.0	29.0
85	8.0	16.0	9.0	30.0	8.0	17.0	9.0	30.0
90	10.0	17.0	10.0	32.0	9.0	18.0	10.0	32.0
95	12.0	18.0	13.0	35.0	11.0	18.0	12.0	34.7
99	16.0	20.0	16.4	41.4	16.9	20.0	16.0	39.0

### Cisgender bisexual plus women

Among the 507 participants who identified as cisgender bisexual plus women, the mean age was 31.9 (*SD* = 9.6, range 18–71) years and the mean BMI was 29.0 (*SD* = 8.4) kg/m^2^ (Table 1). Among cisgender bisexual plus women, 85.3% identified as White, 3.1% as Asian or Pacific Islander, 2.9% as Black, 0.2% as Native American, 1.9% as multiracial, and 6.5% as another race. In addition, 5.9% of the participants identified themselves as Hispanic/Latino/a. Among cisgender bisexual plus women, 74.4% had a college degree or higher. Overall, 3.0% of the participants reported having ever been told by a healthcare provider that they had BDD and none reported having been told that they had MD. Norms for the MDDI among cisgender bisexual plus women are presented in Table 3.

### Comparisons between cisgender lesbian women and cisgender bisexual plus women

In unadjusted linear regression analyses, no significant differences in the MDDI total score (B = 0.02, 95% CI -0.01-0.05, *p* = 0.230) or the subscales (DFS [B = 0.00, 95% CI -0.03-0.04, *p* = 0.855], AI [B = 0.27, 95% CI -0.23-0.77, *p* = 0.292], and FI [B = 0.01, 95% CI -0.04-0.06, *p* = 0.580]) were observed for cisgender bisexual plus women compared to cisgender lesbian women.

In linear regression analyses adjusted for sociodemographics, no significant differences in the MDDI total score (B = 0.00, 95% CI -0.03-0.03, *p* = 0.778) or the subscales (DFS [B = − 0.02, 95% CI -0.05-0.02, *p* = 0.398], AI [B = 0.19, 95% CI -0.28-0.65, *p* = 0.432], and FI [B = 0.03, 95% CI -0.05-0.05, *p* = 0.894]) were observed for cisgender bisexual plus women compared to cisgender lesbian women.

## Discussion

Sexual minority populations have been mostly neglected in the MD literature, despite evidence of greater levels of body dissatisfaction and drive for muscularity among individuals with sexual minority identities [13–16]. Further, most prior validation studies of the MDDI did not assess and/or report sexual orientation data for the samples [5, 9, 23, 24, 26, 33–35]. In this study, we provide the first norms for the MDDI in community samples of cisgender sexual minority men and women. Specifically, we examined the MDDI in cisgender gay men, cisgender bisexual plus men, cisgender lesbian women, and cisgender bisexual plus women. Notably, we did not find significant differences in MDDI scores between cisgender gay men compared to cisgender bisexual plus men, or between cisgender lesbian women compared to cisgender bisexual plus women in unadjusted or adjusted comparisons. This study contributes to the literature on MD among sexual minorities, providing norms for the MDDI and informing a broader understanding of the nature and degree of MD symptoms in cisgender sexual minority men and women from the broad U.S. community.

Compared to the initial MDDI validation study in a selected sample of heterosexual male weightlifters (presumably cisgender men) [20], our community sample of cisgender sexual minority men reported qualitatively higher MDDI total scores (26.4–27.4 vs 18.8), DFS subscale scores (9.0–9.9 vs. 7.5), and AI subscale scores (11.1–11.5 vs 6.12) but similar FI subscale scores (6.1–6.4 vs. 6.4). This is noteworthy, as we would expect even greater differences in the scores found here versus those from a general community sample of heterosexual men.

The unique factors that may contribute to the development, maintenance, or exacerbation of MD symptoms among sexual minorities require further research. Minority stress theory, which suggests that stigma, prejudice, and discrimination experienced by sexual minorities may lead to health disparities [36], may be an important consideration with regard to the experience of MD symptoms in sexual minority individuals. For example, individuals experiencing minority stress may experience psychological (e.g.*,* depressive or anxious symptoms, low self-esteem) or behavioral disturbances (e.g.*,* pathological exercise, eating pathology) that could contribute to elevated risk for muscularity-oriented psychopathology, including MD [37–39]. Further, sexual minority people may experience intra-minority stress, which represents unique status-based competitive and appearance pressures emerging from within sexual minority communities [40, 41]. Bisexual plus people may also experience biphobia, which represents the additional stressors from people with monosexual orientations, including gay, lesbian, or heterosexual people [42]. Despite the additional stressors experienced by bisexual plus people, we did not see significant differences in the MDDI between cisgender bisexual plus men and cisgender gay men, or between cisgender bisexual plus women and cisgender lesbian women.

### Strengths/limitations

Strengths of this investigation include the focus on understudied populations in the MD literature, the large sample sizes across most groups, and the focus on a widely used and well-validated measure of MD symptoms. However, certain limitations also should be noted. Overall, the samples were highly educated, predominantly White, and recruited online, which may limit generalizability. The sample of bisexual plus men was also notably smaller than the other samples. Further, we defined bisexual plus as people who identified as bisexual, pansexual, and/or polysexual, which may obscure potential differences across these groups. Given that the construct of MD was originally conceptualized predominantly in reference to cisgender men, and in light of the different sociocultural body ideals for men and women, the MDDI may be less applicable to cisgender women; however, we made a slight adaptation for cisgender women to specify “chest (muscle)” so as to not confuse “chest” with breast size [30]. Nevertheless, better understanding the construct and measurement of MD in cisgender women versus cisgender men will require further research. Finally, although gender minorities are also understudied in the MD literature, this study focused on cisgender sexual minorities; investigating the MDDI in gender minorities will therefore be an important area for future research.

## Implications and conclusions

This study has several potential clinical and research implications. We report for the first time MDDI norms among cisgender gay men, cisgender bisexual plus men, cisgender lesbian women, and cisgender bisexual plus women. Given that health differences and disparities by sexual orientation are increasingly recognized, establishing descriptive data about MD may enable clinicians and researchers to interpret MDDI scores among these understudied populations. Clinicians should be aware that cisgender sexual minority men and women may experience MD symptoms and should consider assessing for these symptoms and related behaviors when appropriate. In particular, sexual minority community centers and organizations may consider raising awareness and providing support for MD. Future research will be needed to examine the MDDI in clinical samples of sexual minorities diagnosed with MD, and investigate how sociodemographic factors such as race/ethnicity, age, and socioeconomic status may influence the nature and degree of MD symptoms among cisgender sexual minority men and women. Research of this kind will help inform the development of targeted interventions to address MD symptoms in these underserved populations.

## Supplementary Information

**Additional file 1.** Explanation of Classification of Participants.

## Data Availability

Data from The PRIDE Study may be accessed through an Ancillary Study application (details at pridestudy.org/collaborate).

## Abbreviations

AI: Appearance Intolerance subscale
BMI: Body mass index
DFS: Drive for Size subscale
FI: Functional Impairment subscale
M: Mean
MD: Muscle Dysmorphia
MDDI: Muscle Dysmorphic Disorder Inventory
PRIDE Study: Population Research in Identities and Disparities for Equality (PRIDE) Study
SD: Standard deviation
