# Supplementary material for: Community norms of the Muscle Dysmorphic Disorder Inventory (MDDI) among cisgender sexual minority men and women
Source: BMC Psychiatry. 2021 Jun 8;21:297. doi: 10.1186/s12888-021-03302-2 (PMC8186088; doi:10.1186/s12888-021-03302-2)
Supplement: Supplementary file 1 — Additional file 1. Explanation of Classification of Participants. [file 12888_2021_3302_MOESM1_ESM.docx]

| Appendix 1. Explanation of Classification of Participants | | | |
| --- | --- | --- | --- |
| Sample | Sex assigned at birth | Gender identity | Sexual orientation |
| Cisgender gay man (N=1090) | male | man (exclusively) | gay (exclusively) |
| Cisgender bisexual plus man (N=100) | male | man (exclusively) | bisexual (n=82), pansexual (n=32), and/or polysexual (n=0) |
| Cisgender lesbian woman (N=563) | female | woman (exclusively) | gay/lesbian (exclusively) |
| Cisgender bisexual plus woman (N=507) | female | woman (exclusively) | bisexual (n=388), pansexual (n=221), and/or polysexual (n=3) |

Note: Cisgender gay men exclusively indicated “man” as their gender identity and “gay” as their sexual orientation. Cisgender lesbian women exclusively indicated “woman” as their gender identity and “lesbian” and/or “gay” as their sexual orientation. Participants who reported multiple gender identities were excluded.
